# Supplementary material for: Laser scribed proton exchange membranes for enhanced fuel cell performance and stability
Source: Nat Commun. 2024 Dec 30;15:10811. doi: 10.1038/s41467-024-55070-8 (PMC11685907; doi:10.1038/s41467-024-55070-8)
Supplement: Supplementary file 1 — Supplementary information [file 41467_2024_55070_MOESM1_ESM.pdf]

Supporting information

Laser Scribed Proton Exchange Membranes for Enhanced Fuel Cell Performance and Stability

Jianuo Chen[1,2], Xuekun Lu[3], Lingtao Wang[4], Wenjia Du[1,5], Hengyi Guo[4], Max Rimmer[4], Heng Zhai[2], Yuhan Liu[1], Paul R. Shearing[5], Sarah J. Haigh[4], Stuart M. Holmes[2], Thomas S. Miller[1*]

[1] Electrochemical Innovation Lab, Department of Chemical Engineering, University College London, London, WC1E 7JE, UK.

[2] Department of Chemical Engineering, University of Manchester, Oxford Road, Manchester, M13 9PL, UK

[3] School of Engineering and Materials Science, Queen Mary University of London, London, UK

[4] Department of Materials, University of Manchester, Oxford Road, Manchester, M13 9PL, UK

[5] Department of Engineering Science, University of Oxford, Oxford, OX1 3PJ, UK

Email: t.miller@ucl.ac.uk

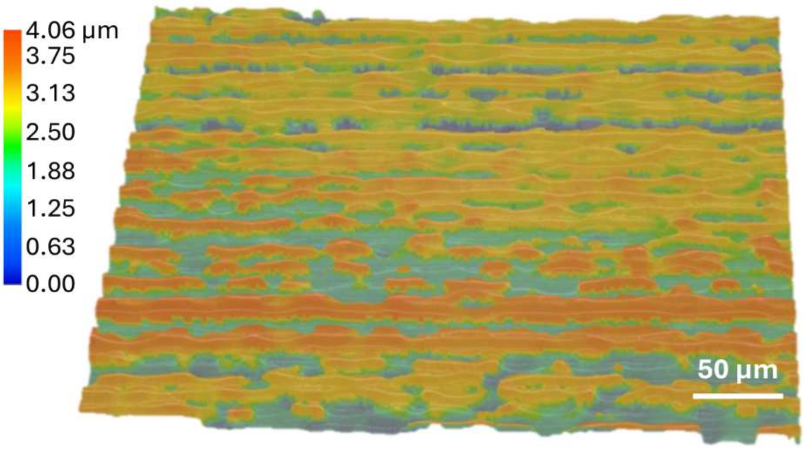

Fig. S 1. Optical microscopy of laser-induced PBI membrane with depth profile.

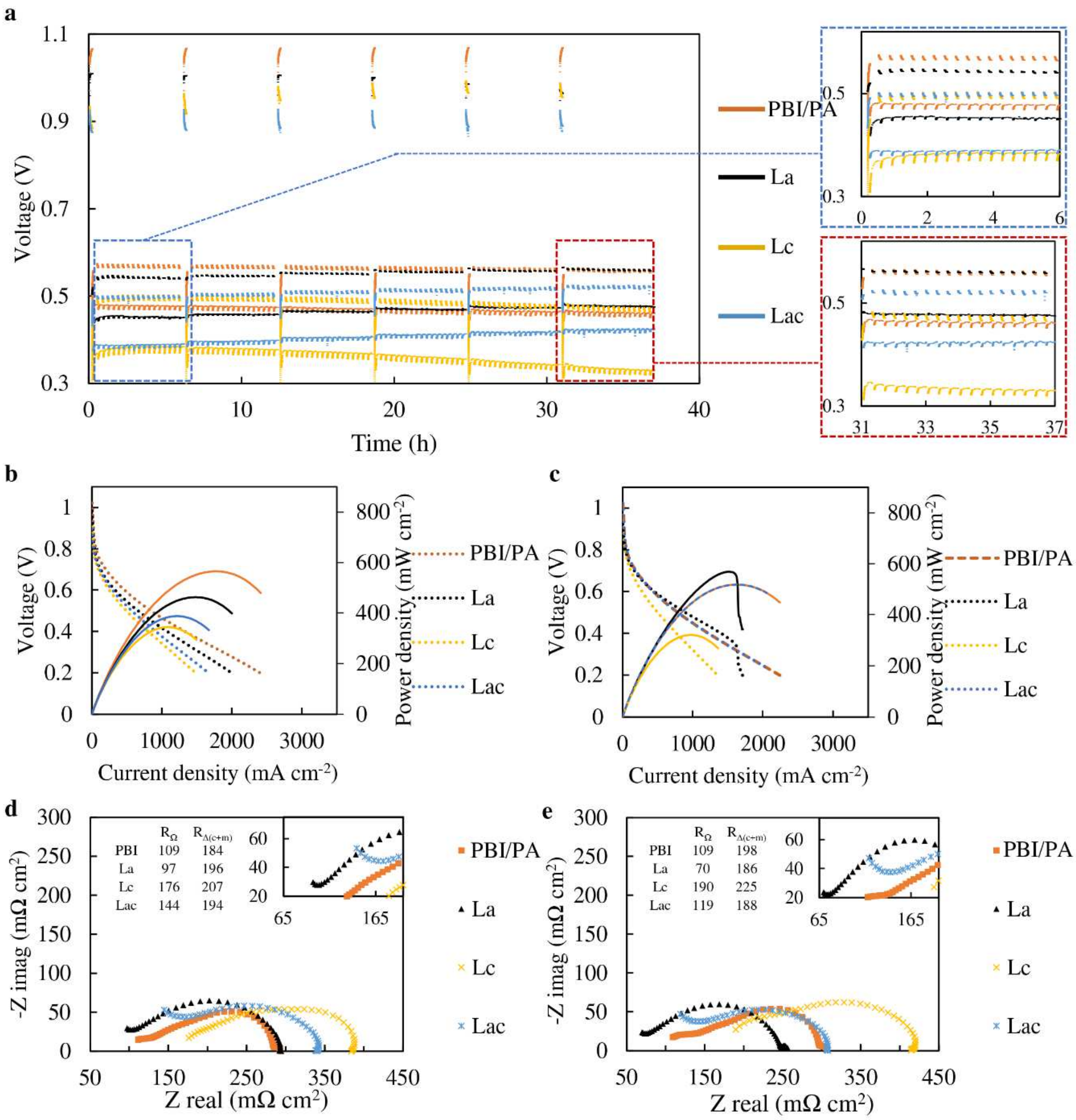

Fig. S 2. Electrochemical characterization of MEAs based on different membranes. a AST. **b** polarization curves before AST. **c** polarization curves after AST, **d** EIS curves before AST, **e** EIS curves after AST

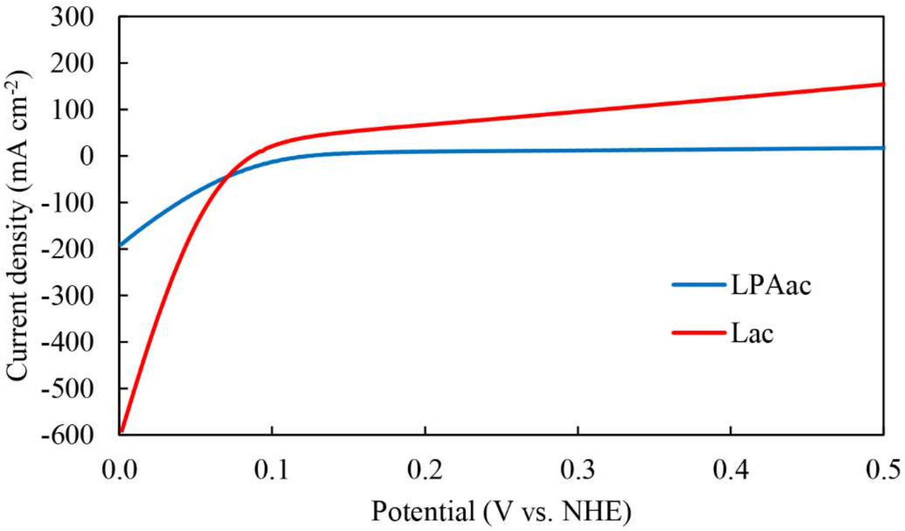

Fig. S 3. LSV curves of LPAac and Lac.

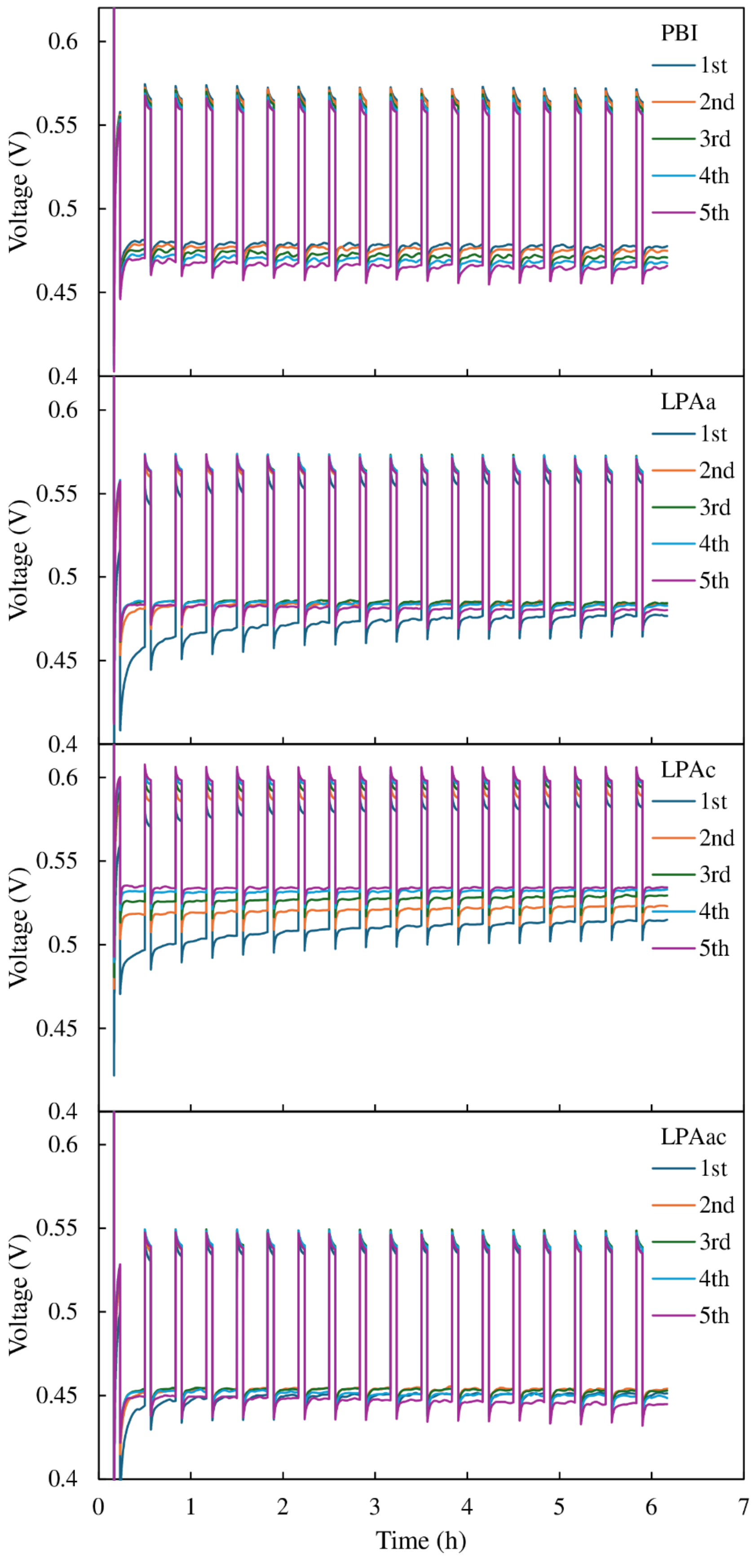

Fig. S 4. Stacked AST plots from the first cycle to the fifth cycle (repeating chronopotentiometry between 0.6 A cm^{-2} for 4 min and 1 A cm^{-2} for 16 min, running OCV for 10 min every 6 hr)

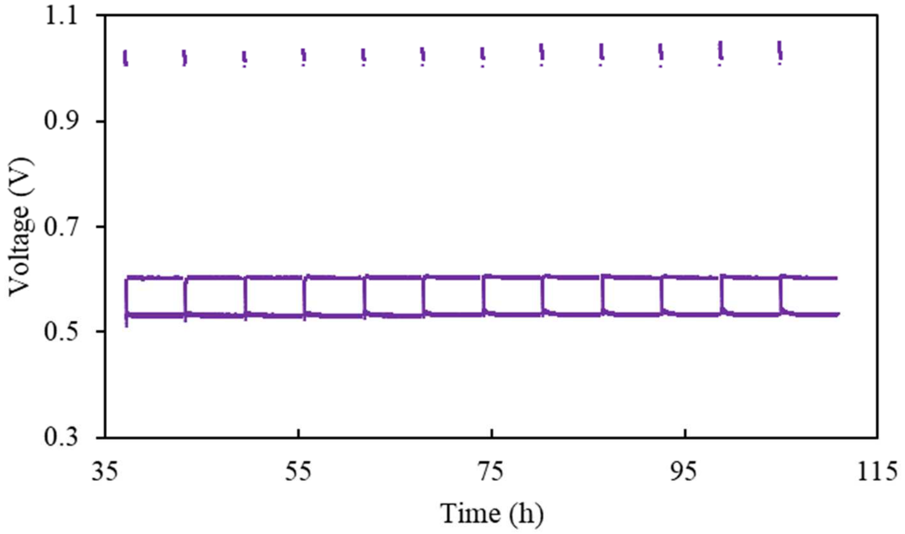

Fig. S 5. Extended AST plots of LPAc, (repeating chronopotentiometry between 0.6 A cm-2 for 4 min and 1 A cm-2 for 16 min, running OCV for 10 min every 6 hr)

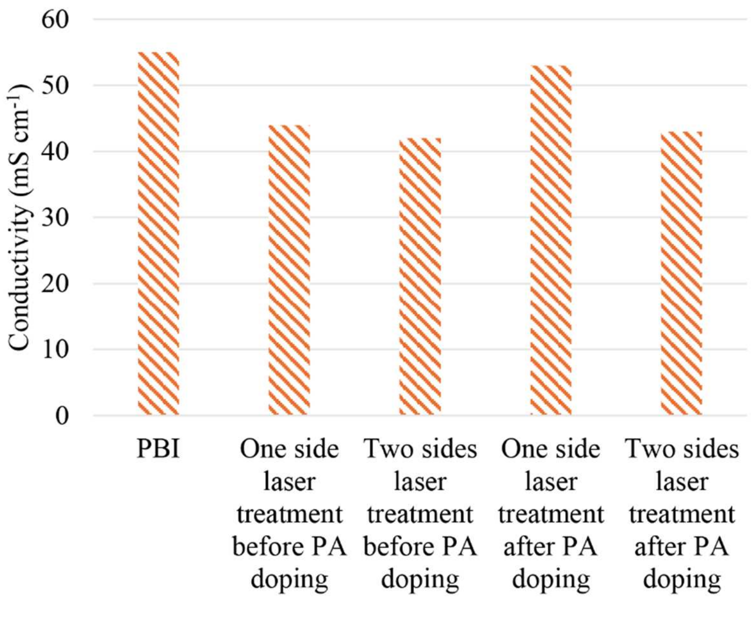

Fig. S 6. Proton conductivity of PA doped PBI membrane and laser-treated PBI membranes.

a

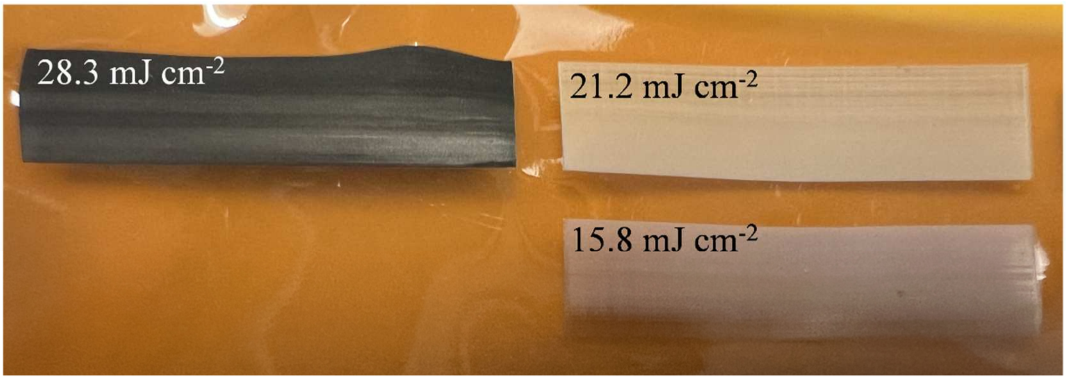

b

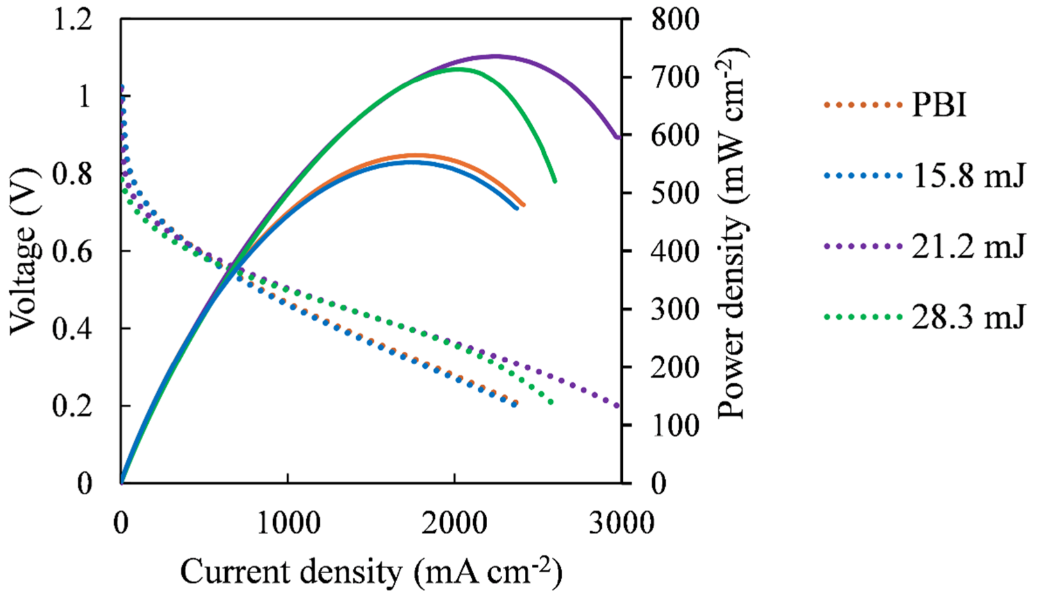

Fig. S 7. LPAc with different laser fluences of 15.8 mJ cm^{-2}, 21.2 mJ cm^{-2}, and 28.3 mJ cm^{-2} , a Membrane surface**. b** Polarization curves and power density curves of LPAc 160℃, anode: H_2 (λ=1.2), cathode: O_2 (λ=2.0).

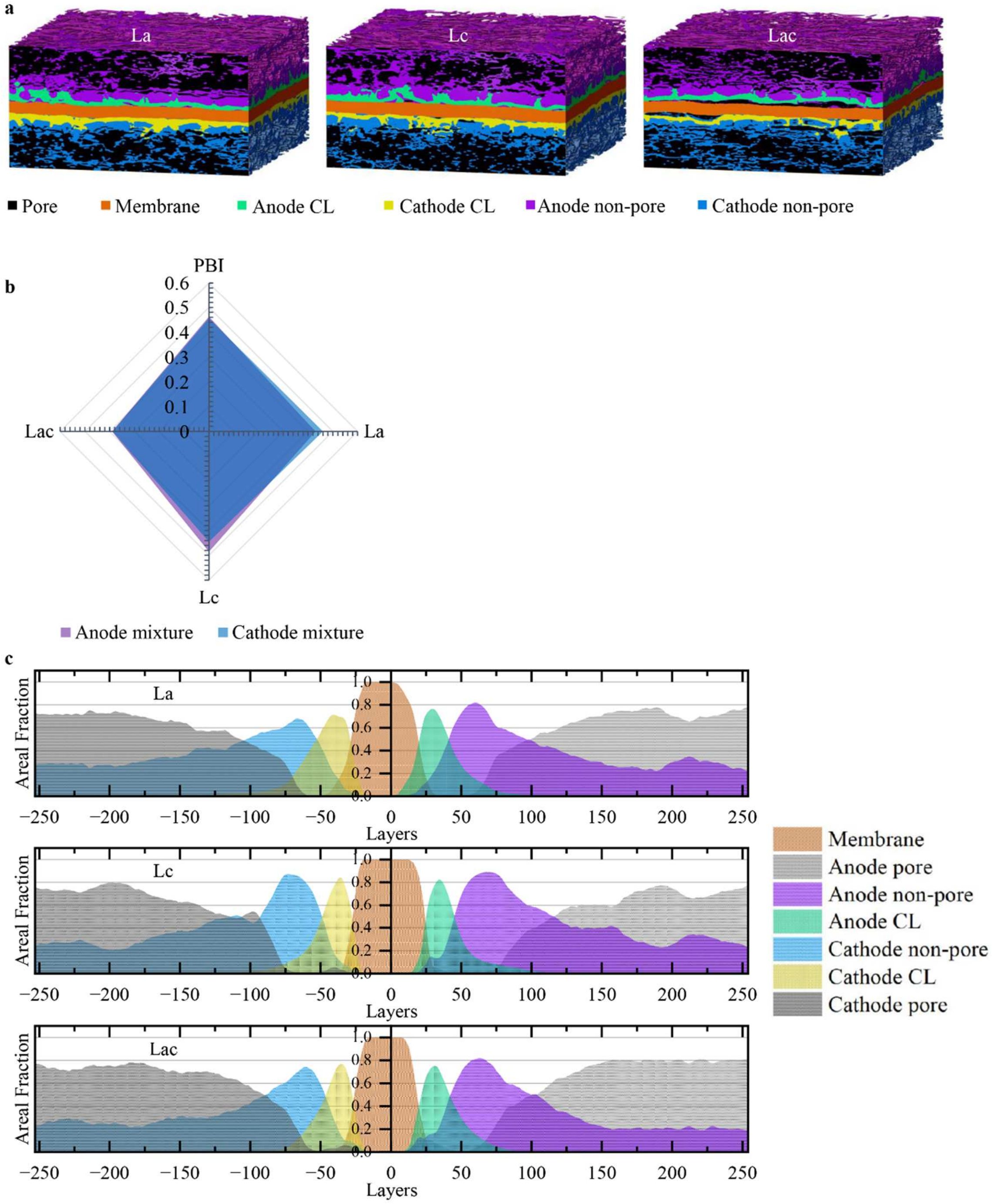

Fig. S 8. The three-dimensional structure and component spatial distribution of MEA. a Volume rendering of segmented MEAs. **b** Electrode average mixture phase volume fraction. **c** Slice-by-slice plots of area fraction of volume rendering in Z direction.

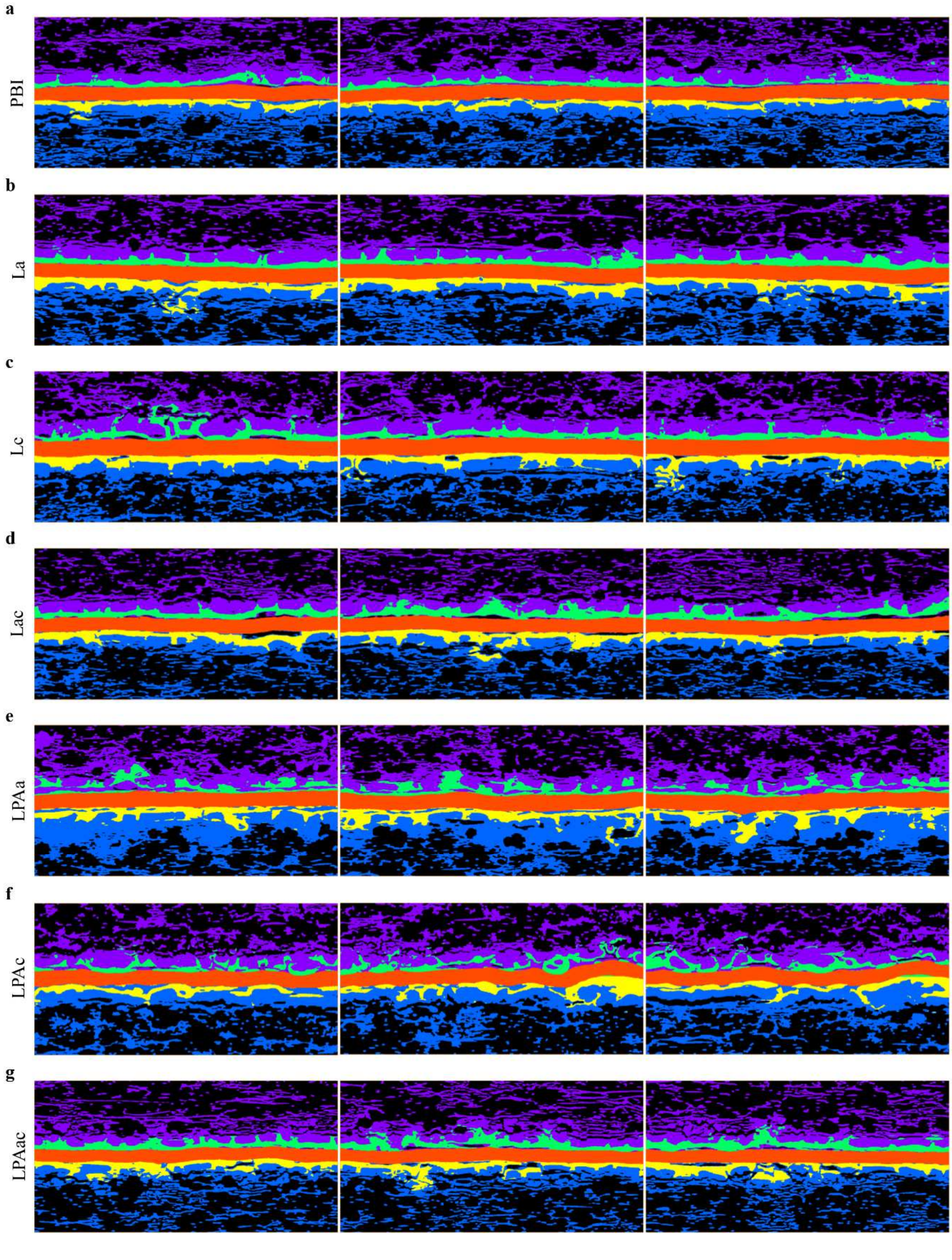

Fig. S 9. Typical segmented orthoslices of different MEAs after AST. a PBI. **b** La. **c** Lc. **d** Lac. **e** LPAa. **f** LPAc. **g** LPAac.

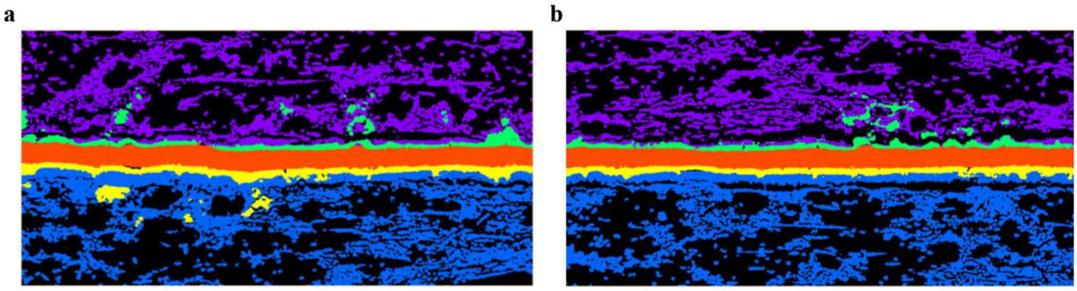

Fig. S 10. Typically segmented orthoslices of different MEAs before AST. a LPAa. **b** LPAc.

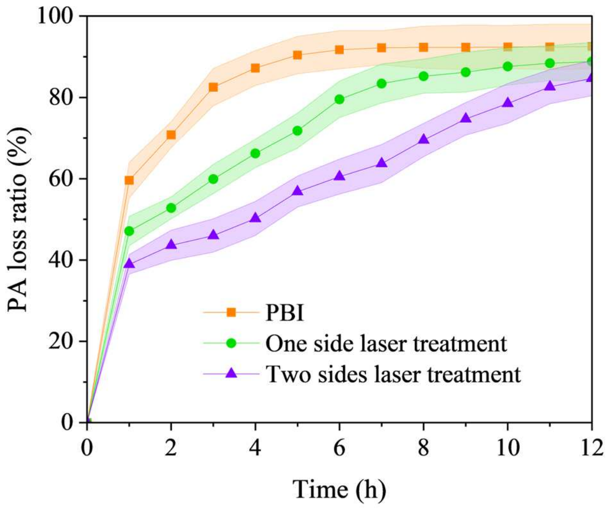

Fig. S 11. Time dependence of weight loss ratio of acid in PBI, one side laser treated PBI and two side laser treated PBI.

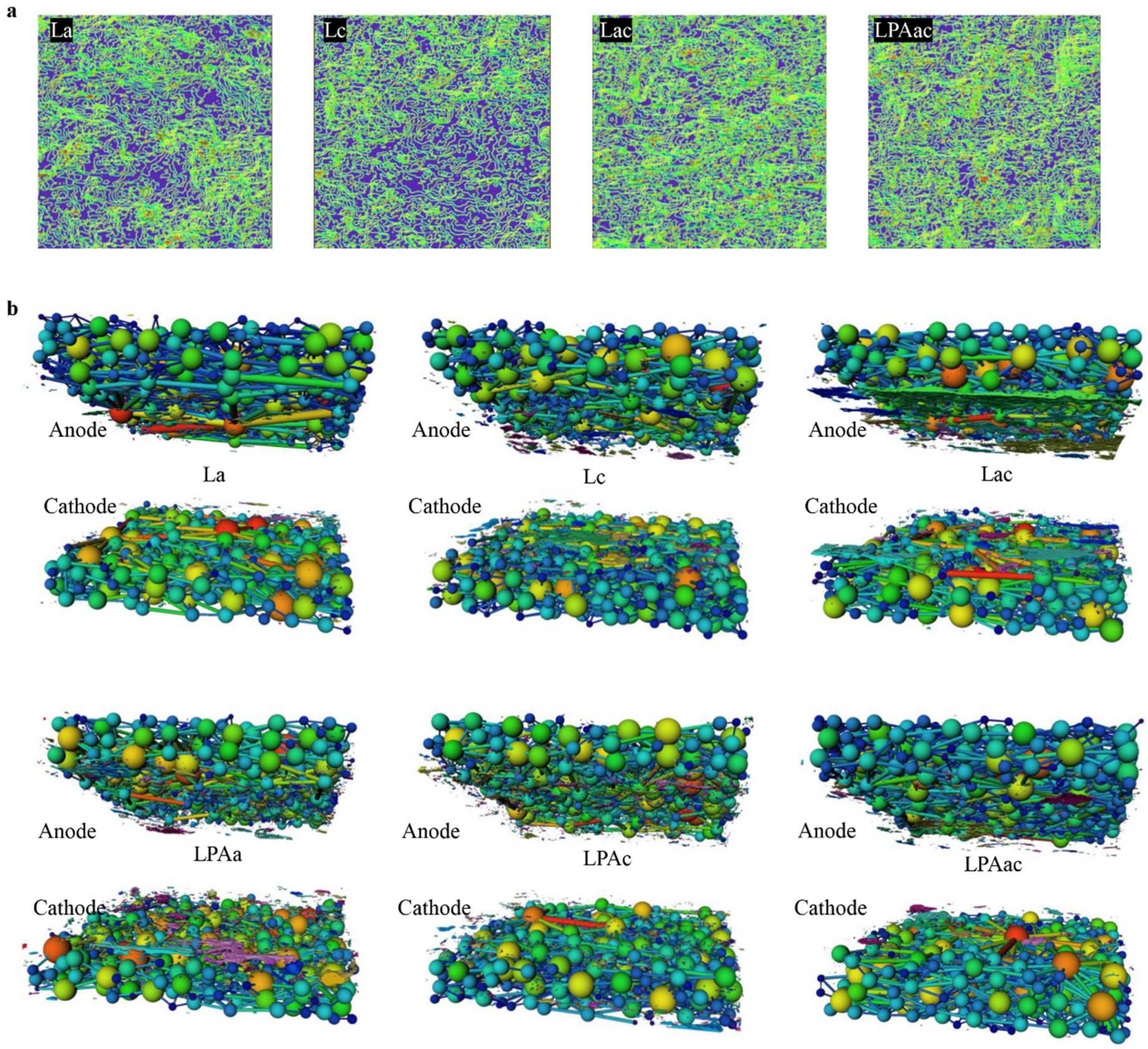

Fig. S 12. Pore structure and spatial distribution in MEAs. a *The curvature distribution plots of the membrane.* **b** *The ball-and-stick model of the electrode pores and throat network, along with the distribution plot of isolated pores.*

Lc has a flat membrane and exhibits a significant number of isolated pores in both the cathode and anode electrodes as shown in Fig. S **6**a. However, the peak of the isolated pore curve for Lc is farther away from the membrane compared to Lac and LPAac. From Fig. S2a, it is also evident that Lc does not have a distinct gap between the membrane and the CL like Lac and LPAac. Therefore, the presence of numerous isolated pores in Lc could be related to the scattered distribution of PA within the electrode.

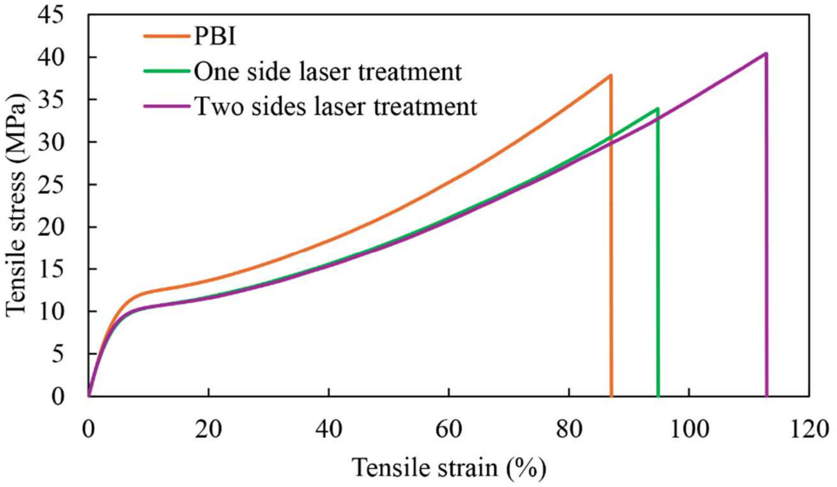

Fig. S 13. Stress–strain curves of PBI and laser induced PA doped PBI membrane.

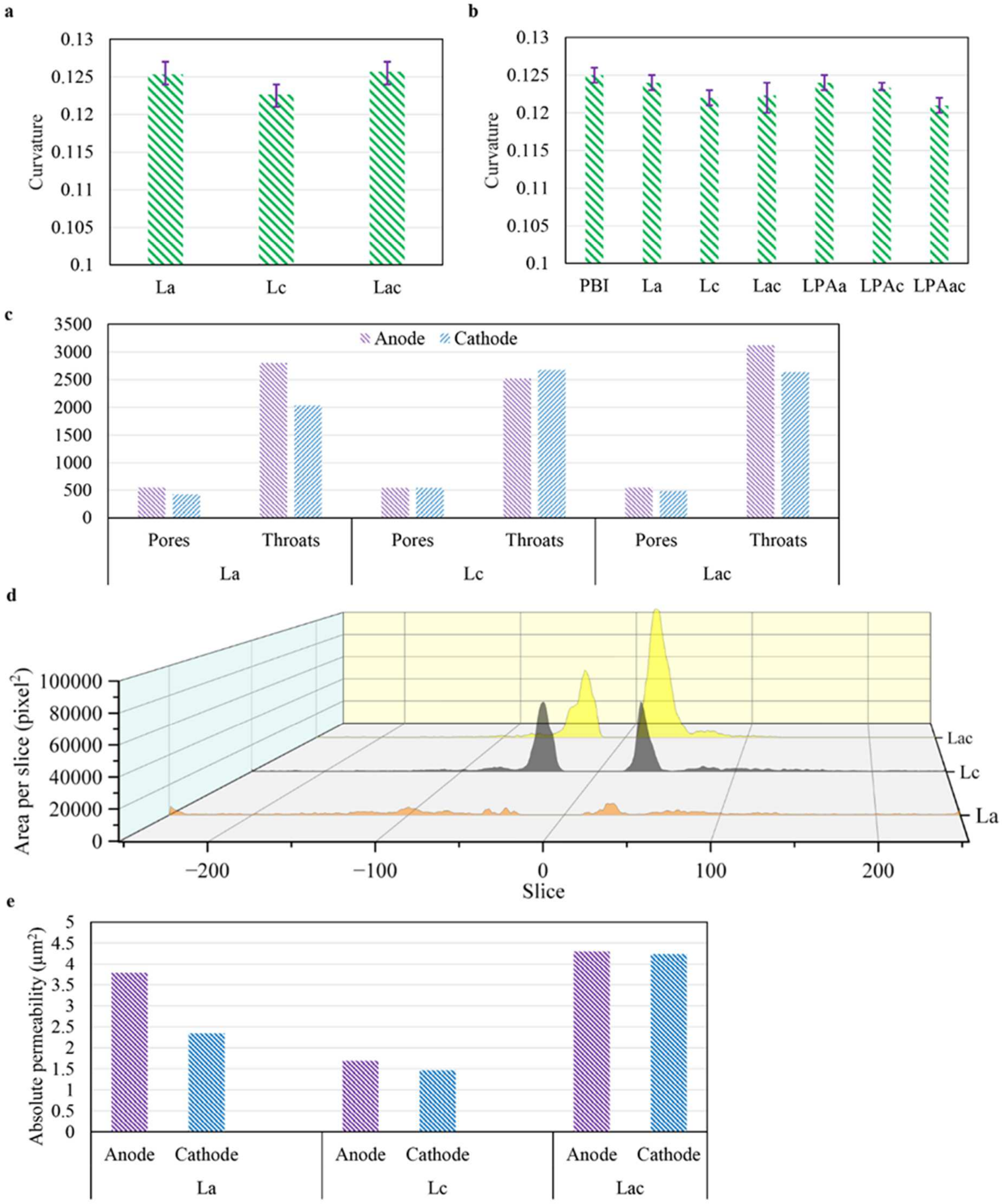

Fig. S 14. Pore structure and spatial distribution in MEAs. a The statistical distribution plot of the average curvature of the membrane after AST. **b** The statistical distribution plot of the average curvature of the membrane before AST. **c** Statistical plots depicting the average number of pores and throats in electrodes across different MEAs after AST. **d** Slice-by-slice plots of isolated pores of different MEAs after AST. **e** statistical plots of absolute permeability for different MEAs' pore region after AST.

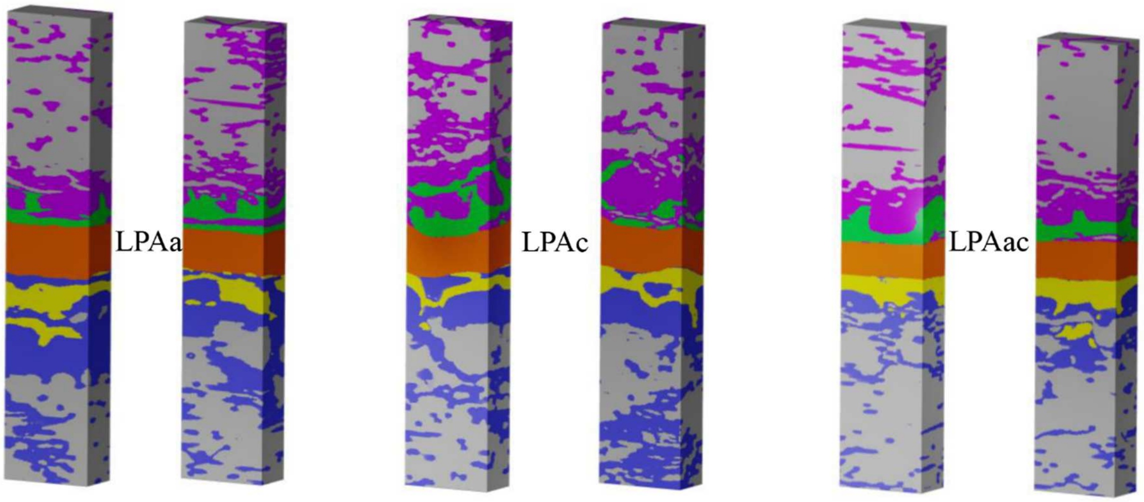

Fig. S 15. MEA models for simulation

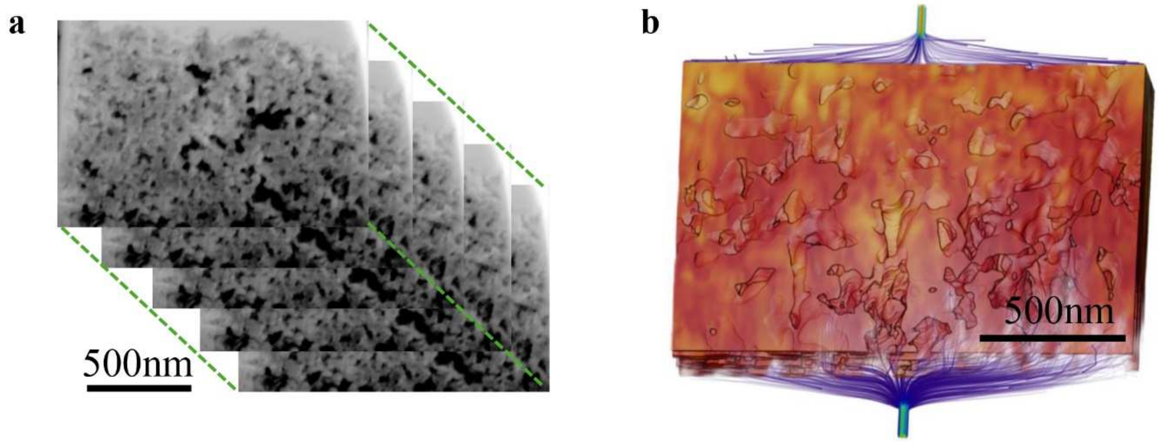

Fig. S 16. 3D structure of CL. a FIB-SEM images of CL**. b** Illuminated streamlines of absolute permeability in the CL.

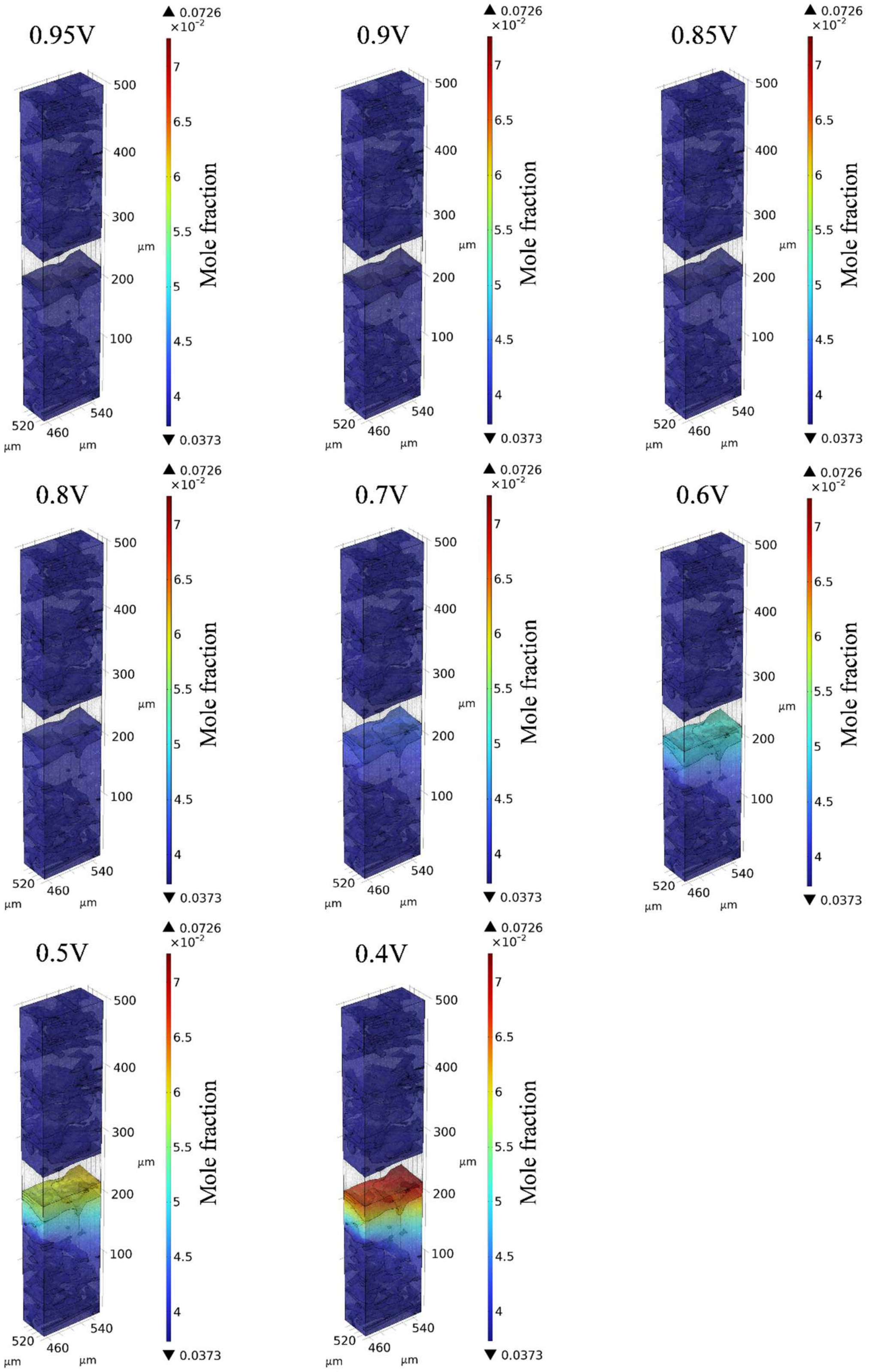

Fig. S 17. Water mole fraction distribution of LPAa at different voltages

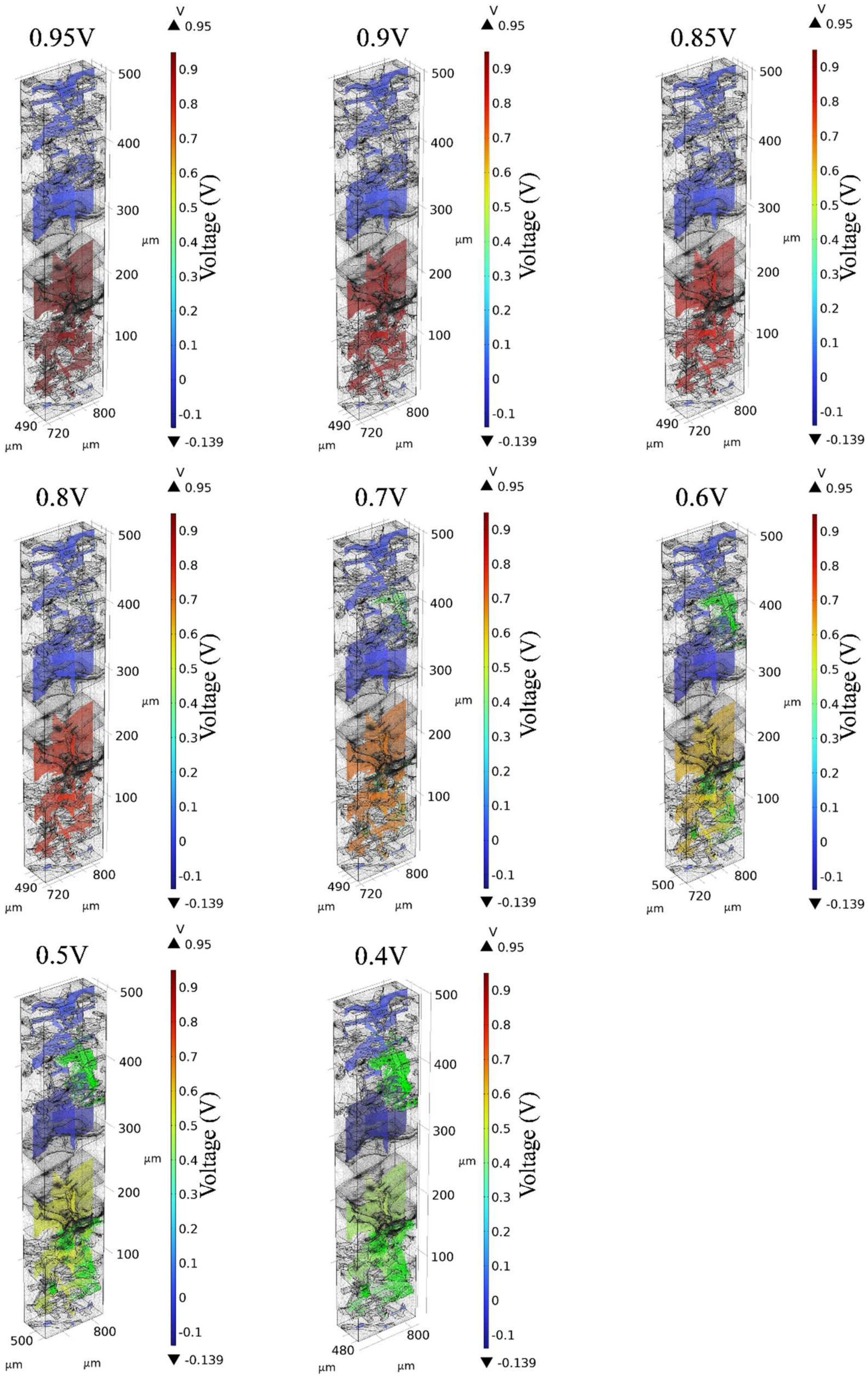

Fig. S 18. Electrode potential distribution with respect to ground (arrow volume: electrode current density vector) at different voltages of LPAc.

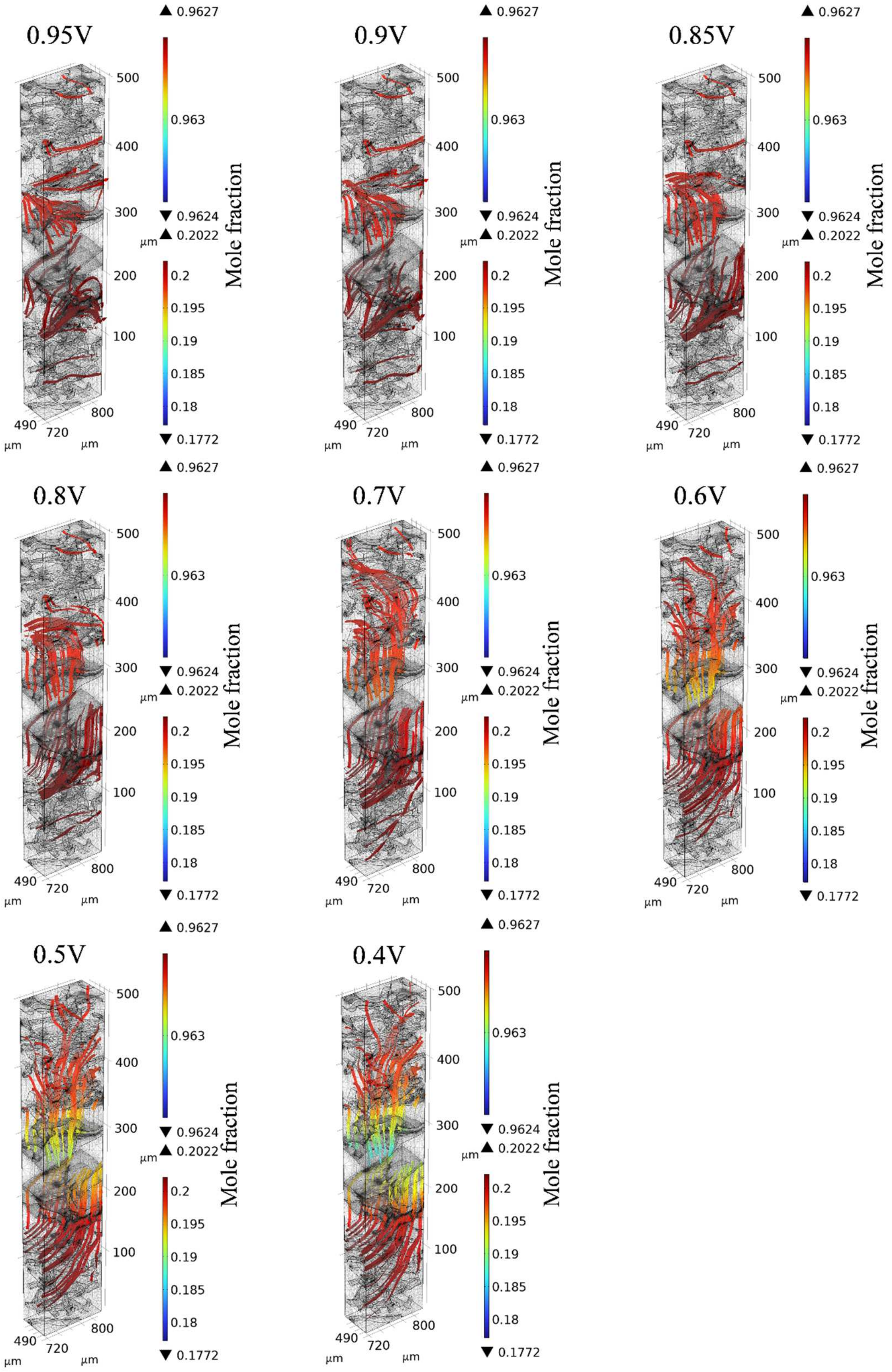

Fig. S 19. Total flux streamline of H_2 and O_2 at different voltages of LPAc.

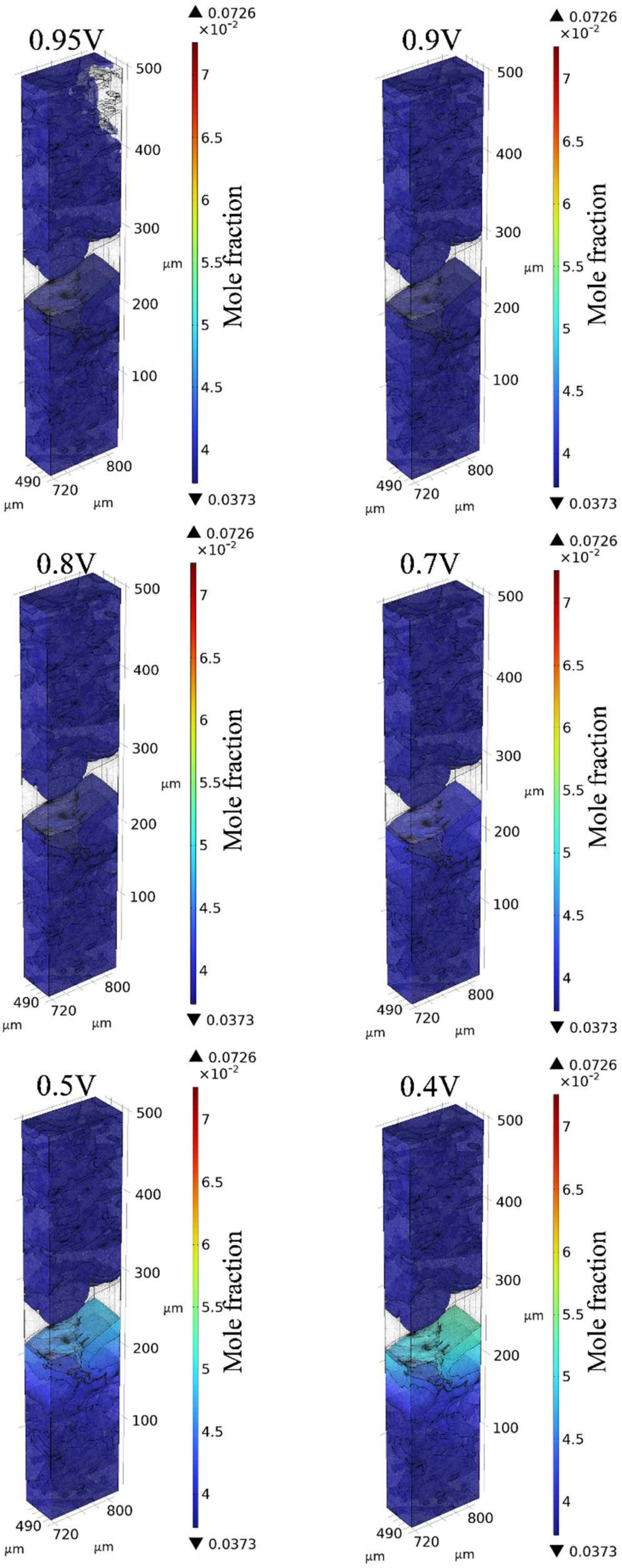

Fig. S 20. Water mole fraction distribution of LPAc at different voltages

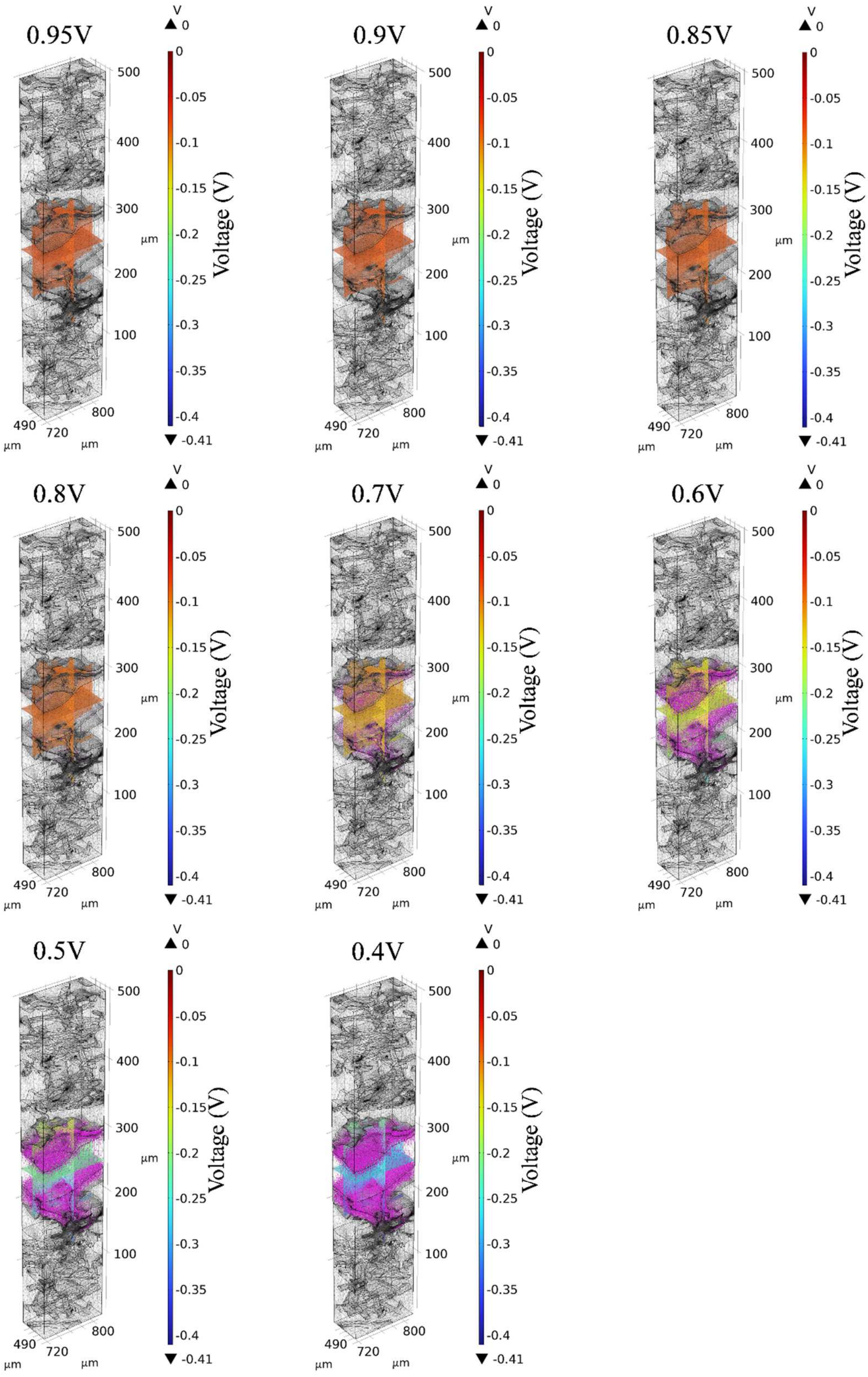

Fig. S 21. Electrolyte potential distribution (arrow volume: electrode current density vector) at different voltages of LPAc.

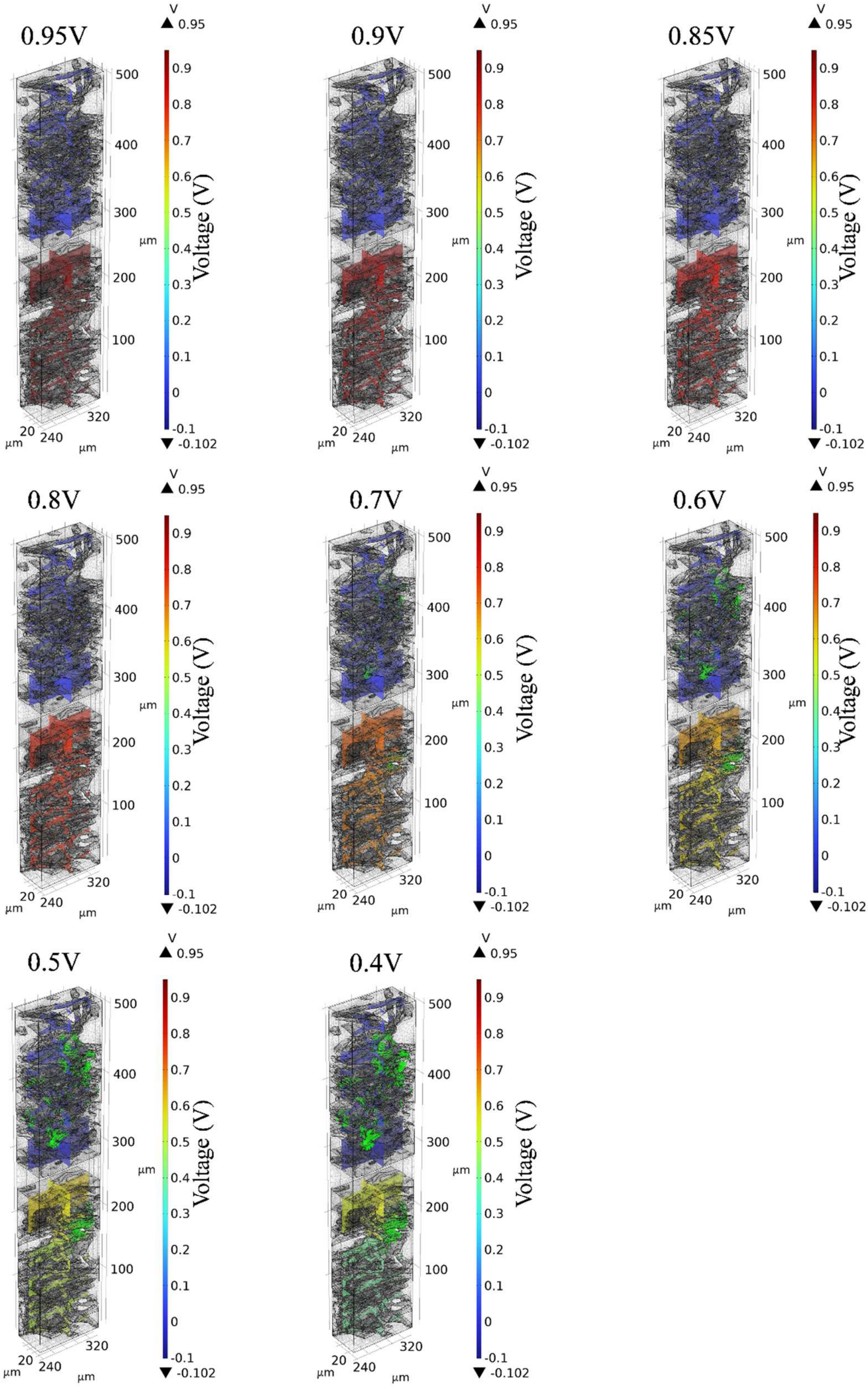

Fig. S 22. Electrode potential distribution with respect to ground (arrow volume: electrode current density vector) at different voltage of LPAac.

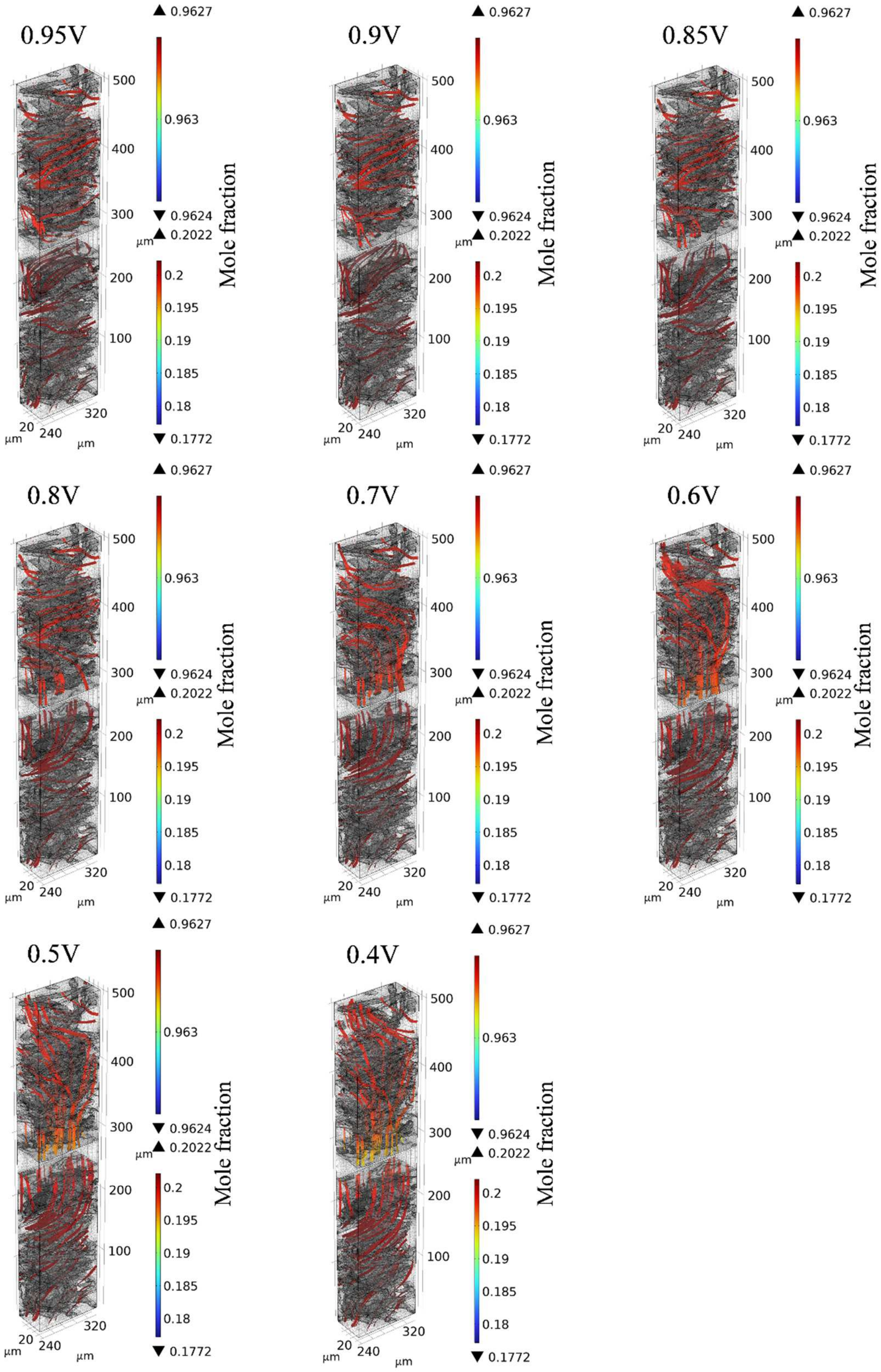

Fig. S 23. Total flux streamline of H_2 and O_2 at different voltages of LPAac.

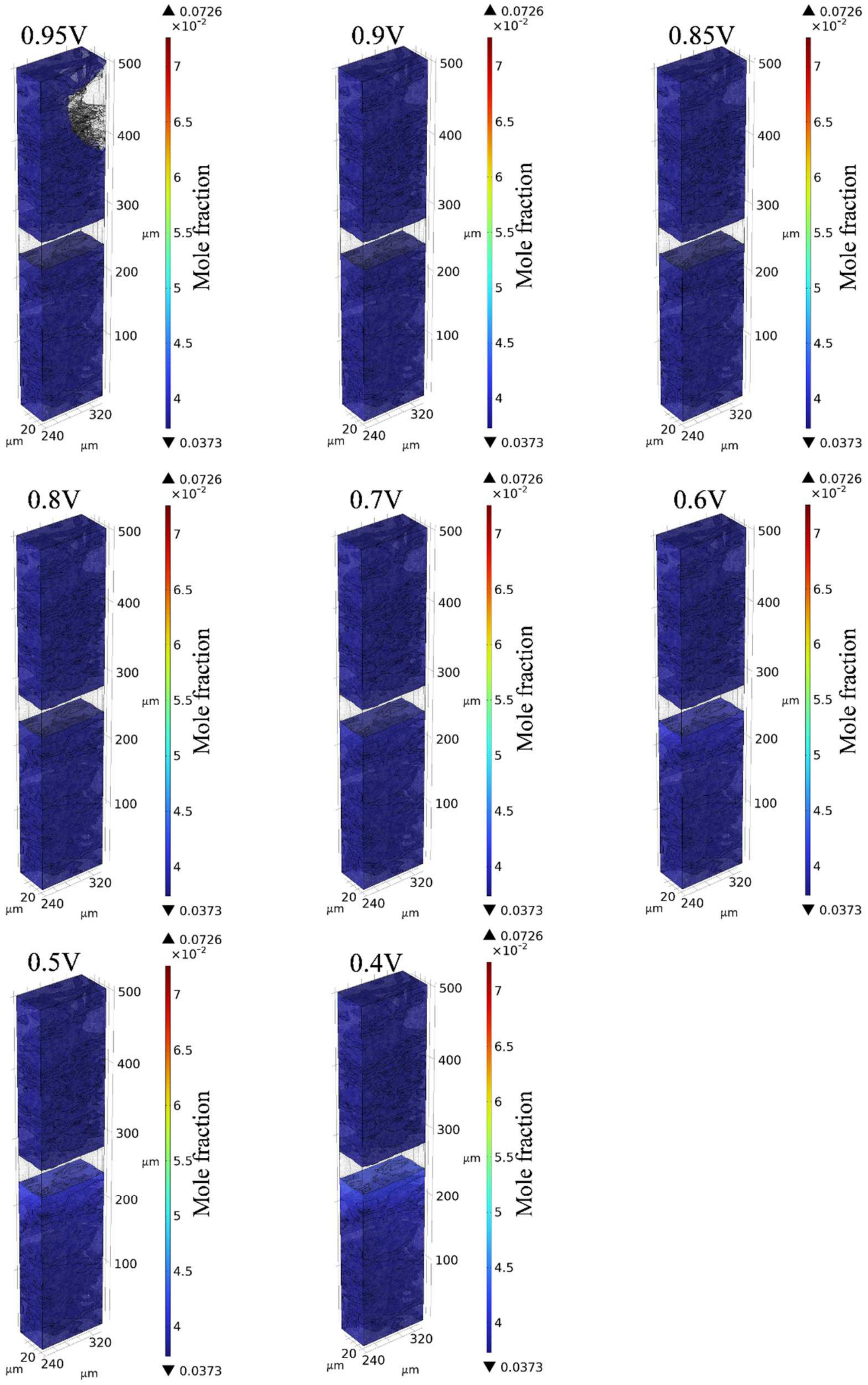

Fig. S 24. Water mole fraction distribution of LPAac at different voltage

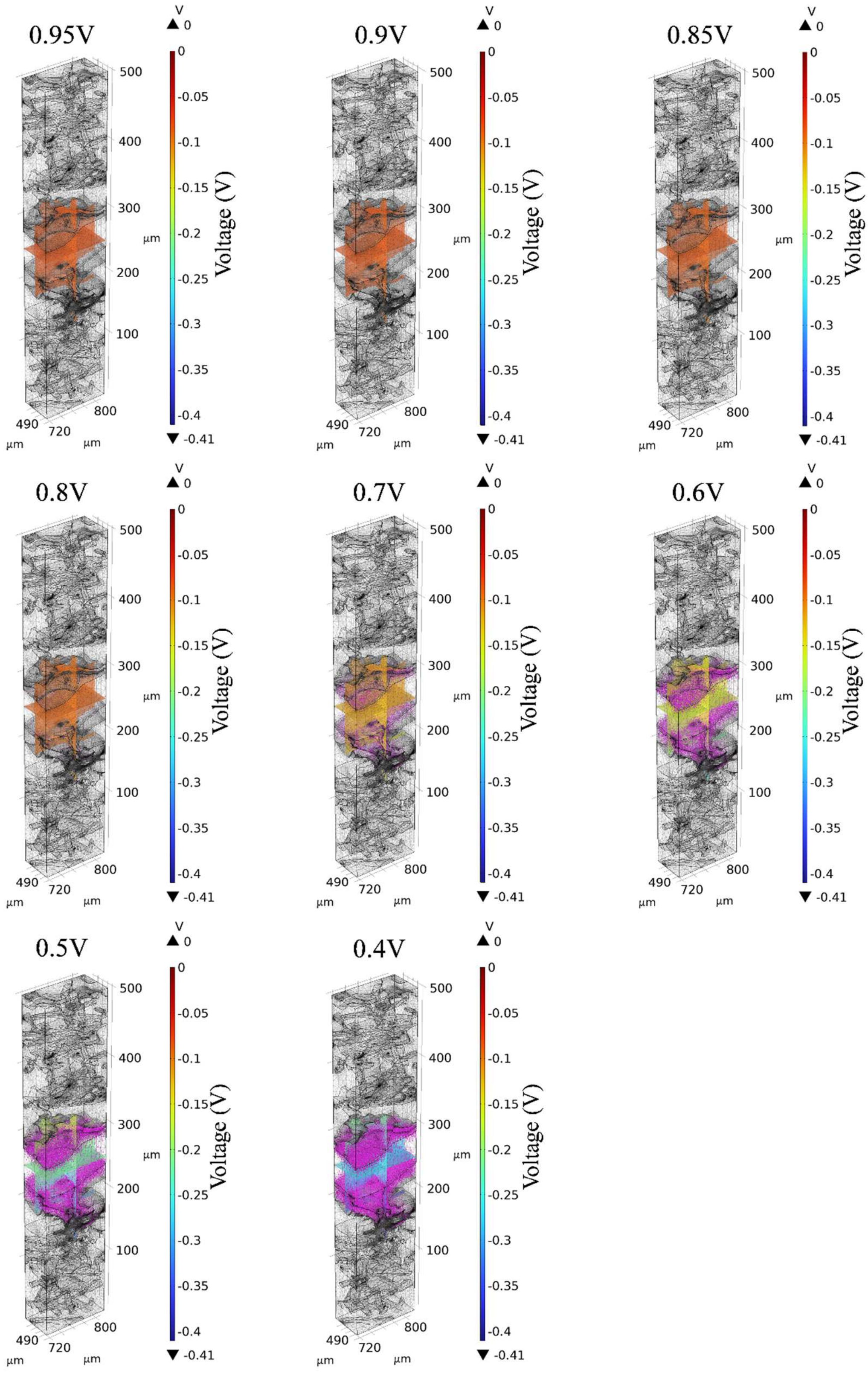

Fig. S 25. Electrolyte potential distribution (arrow volume: electrode current density vector) at different voltage of LPAac.

Table S1. The equivalent circuit and the definition of parameters

Equivalent circuit	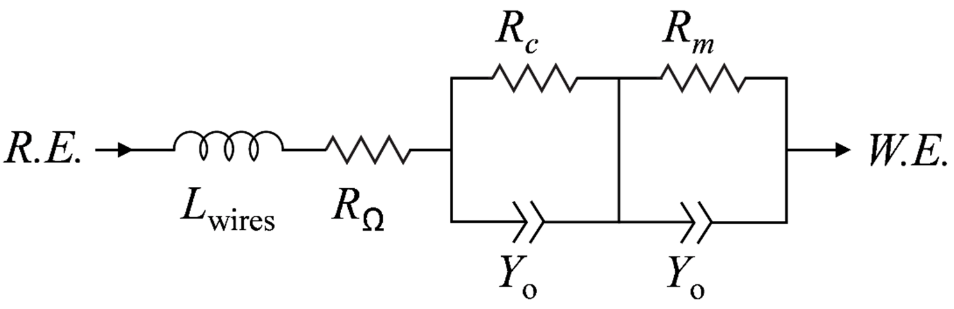
L_{wires} (H)	Inductance of cables
R_{Ω} (ohm)	Ohmic resistance
R_{c} (ohm)	Charge transfer resistance
R_{m} (ohm)	Mass transfer resistance
Y_{o} (S s^{a})	Constant phase element
a	Dimensionless exponent

Table S2. Key parameters for simulations in COMSOL

Name	Value or Expression			Description
	LPAa	LPAc	LPAac	
T	433.15 K			Cell temperature
p_{ref}	1 atm			Reference pressure
σ_m	$5.6\ S\ m^{-1}$	$5\ S\ m^{-1}$	$4.9\ S\ m^{-1}$	Membrane conductivity (from EIS)
$\varepsilon_{gas,gdl}$	based on real 3D structure (Fig. 4)			Gas diffusion layer gas pore volume fraction
$\varepsilon_{gas,mix}$	$=\varepsilon_{gas,gdl}$			Mixture phase gas pore volume fraction
$\varepsilon_{gas,cl}$	$=\varepsilon_{gas,cl}$			Catalyst layer gas pore volume fraction
$\varepsilon_{l,cl}$	$1-\varepsilon_{gas,cl}$			Catalyst layer electrolyte volume fraction
$\kappa_{p,gdl}$	based on real 3D structure (Fig. 4)			Gas diffusion layer permeability
$\kappa_{p,mix}$	$\kappa_{p,gdl}/6.25$			Mixture phase permeability
$\kappa_{p,cl}$	$\kappa_{p,gdl}/6.25$			Catalyst layer permeability
$i_{0,ref,a}$	$1e^{2}(A\ m^{2})$			Reference exchange current density, anode
$i_{0,ref,c}$	$1e^{-3}(A\ m^{2})$			Reference exchange current density, cathode
α_c	1			Transfer coefficient, cathode
T_{hum}	301.15 K			Humidification temperature
p_{vapour}	3781.4 Pa			Water partial pressure
$\chi_{H_2o,in}$	0.037319			Inlet water molar fraction
$\chi_{H_2,in}$	0.96268			Inlet hydrogen molar fraction
$\chi_{O_2,in}$	0.20216			Inlet oxygen molar fraction
λ_a	1.2			Anode stoichiometry
λ_c	2			Cathode stoichiometry

Hydrogen fuel cell interface:

Maxwell-Stefan equation to study fluxes of mass fraction

$$\nabla\cdot\left(-\rho\omega_i\sum_j\left(D_{ij}\nabla x_j+\left(x_j-\omega_j\right)\frac{\nabla p}{p}\right)+\rho\omega_i u\right)=0$$

D_{ij} : binary diffusivity, m^2s^{-1}

ω : mass fraction

ρ : fluid density, $kg\ m^{-3}$

p : pressure, Pa

u : velocity, $m\ s^{-1}$

The binary diffusion coefficient can be determined using an empirical equation derived from the principles of kinetic gas theory.

$$D_{ij} = \frac{10^{-3} \cdot T^{1.75}}{p\left[(\sum_k V_{ki})^{\frac{1}{3}} + (\sum_k V_{kj})^{\frac{1}{3}}\right]^2} \left(\frac{1}{M_i} + \frac{1}{M_j}\right)^{\frac{1}{2}}$$

$\sum_k V_k$: molar diffusion volume

M: molar mass, $kg\ mol^{-1}$

Free and porous media flow interfaces and reacting flow muliphysics.

Navier-stokes equation to describe momentum transfer

$$\rho(u \cdot \nabla)u + \nabla p - \nabla \cdot \eta(\nabla u + (\nabla u)^T) = 0$$

η : fluid viscosity, $kg\ m^{-1} s$

Darcy's Law added to Navier-stokes equation to study the velocity distribution

$$\rho(u \cdot \nabla)u + \nabla p - \nabla \cdot \eta(\nabla u + (\nabla u)^T) = -\frac{\eta}{\kappa_p} u$$

κ_p : permeability, m^2

The flow within porous media is regulated by a blend of the continuity equation and the momentum equation, collectively constituting the Brinkman equations:

$$\frac{\partial}{\partial t}(\varepsilon_p \rho) + \nabla \cdot (\rho u) = Q_m$$

$$\frac{\rho}{\varepsilon_p}\left(\frac{\partial u}{\partial t} + (u \cdot \nabla)\frac{u}{\varepsilon_p}\right) = -\nabla p + \nabla \cdot \left[\frac{1}{\varepsilon_p}\left\{\mu(\nabla u + (\nabla u)^T) - \frac{2}{3}\mu(\nabla \cdot u)I\right\}\right] - \left(k_p{}^{-1}\mu + \frac{Q_m}{\varepsilon_p^2}\right)u + Force$$

μ : dynamic viscosity of the fluid, $kg\ m^{-1} s$

ε_p : porosity

Q_m : mass source or sink, $kg\ (m^2 \cdot s^2)^{-1}$
