# Supplementary material for: Laser scribed proton exchange membranes for enhanced fuel cell performance and stability
Source: Nat Commun. 2024 Dec 30;15:10811. doi: 10.1038/s41467-024-55070-8 (PMC11685907; doi:10.1038/s41467-024-55070-8)
Supplement: Supplementary file 3 — Description of Additional Supplementary Files [file 41467_2024_55070_MOESM3_ESM.pdf]

<u>Description of Additional Supplementary Files</u>

Supplementary Movie 1: Visualisation simulation of gas distribution potential, current density and water distribution of LPAa at different voltage
